# Regulation of Ferroptosis by Transcription Factor E2F1 and RB

**DOI:** 10.21203/rs.3.rs-2493335/v1

**Published:** 2023-01-30

**Authors:** Nishanth Kuganesan, Samkeliso Dlamini, Viranga LM Tillekeratne, William R Taylor

**Affiliations:** University of Toledo; University of Toledo; University of Toledo; University of Toledo

**Keywords:** ferroptosis, SLC7A11, E2F, retinoblastoma protein (RB), cyclin E, cyclin dependent kinase (CDK)

## Abstract

Tumor suppressor RB binds to E2F family proteins and modulates cell cycle progression. Cyclin dependent kinases (CDK) regulate the interaction of RB/E2F by phosphorylating RB. Previously, we have revealed that CDK2, RB and E2F inhibit ferroptosis. Ferroptosis is a non-apoptotic, iron-dependent form of cell death characterized by toxic lipid peroxidation. Here we provide evidence that CDK2 suppresses ferroptosis through phosphorylation of RB. We approach this question by overexpressing WT-RB or a mutant RB that cannot be phosphorylated by CDKs (RBΔCDK) along with CDK2/cyclinE followed by analysis of ferroptosis. We also observed that E2F1 regulates of both pro and anti-ferroptotic proteins including ALOX5, MYC SLC7A11, ATF4, and GPX4 and finally renders a net inhibitory role in ferroptosis. Interestingly, we also found a cell type dependent compensatory effect of E2F3 upon E2F1 depletion. This compensatory effect resulted in no change of ferroptotic target genes after E2F1 knock down in an osteosarcoma cell line. Taken together, our study reveals that cancer cells protect themselves from ferroptosis through cell cycle regulatory proteins.

## Introduction

The E2F and RB families are critical regulators of cell cycle progression. Tumor suppressor proteins including p53, p21 and p16INK in turn regulate E2F/RB. Many cancers develop as a result of bypassing this checkpoint system through mutation or suppression of p53 activity or elevated cyclin dependent kinase (CDK) activity leading to aberrant E2F transactivation [1] [2]. Ferroptosis is a non-apoptotic form of cell death caused by iron-dependent lipid peroxidation [3]. Ferroptosis has gained attention due to rapid killing and more selectively targeting mesenchymal cancer stem cells and drug resistant persistence cells. Our group discovered a small molecule called CETZOLE-1 that induces ferroptosis [4] [5] [6] [7]. Previously we have found that CETZOLE-1 induces ferroptotic cell death by inhibiting system Xc^−^ transporter [5]. SLC7A11 is the catalytic subunit of system Xc^−^ transporter which plays a critical role in maintaining cellular GSH level. Recent experiments demonstrated that CETZOLE-1 covalently binds to GPX4, an enzyme that reduces toxic lipid peroxides to non-toxic alcohols [8]. Therefore, CETZOLE-1 may induce ferroptosis as a dual inhibitor of both system Xc^−^ and GPX4 [5] [8].

Previously, we reported that p53/p21/CDK/RB/E2F pathway proteins differentially regulate ferroptosis [7]. For example, CDK2, RB and E2F1 inhibit ferroptosis [7]. CDKs phosphorylate RB leading to the release of E2F. RB is monophosphorylated during G1 phase and some of the monophosphorylated RB species can still transcriptionally control gene expression [9] [10]. RB species phosphorylated at S811 and T826 have been reported to modulate oxidative phosphorylation genes [10]. Therefore, one aim of our current study is to test if RB phosphorylation regulates ferroptosis sensitivity.

E2F1 is one of the major transcription factors that controls cellular activity through many different cell cycle dependent and independent targets. Our previous study showed that E2F1 inhibits ferroptosis partially through p21 [7]. Our present study focuses on finding additional targets of E2F1 in ferroptosis. interestingly, we observed that SLC7A11 was upregulated at both the mRNA and protein levels upon E2F1 overexpression in HT1080 (fibrosarcoma) cells. Furthermore, compensatory regulation of E2F family members by E2F1 depends on the cell type and influences the regulation of SLC7A11.

Oncogene Myc regulates proliferation, glycolysis, oxidative phosphorylation, glutaminolysis, fatty acid metabolism and apoptosis [11] [12]. N-Myc regulates ferroptosis by modulating cysteine and glutamine utilization [13] [14]. Furthermore, a recent study has reported that N-Myc binds to the promoter region and transcriptionally activates SLC3A2 [15]. Meanwhile SLC7A11 was upregulated at both protein and mRNA levels, nevertheless no ChIP (chromatin immunoprecipitation) signal was found in the same study [15]. Cysteine is a non-essential amino acid as cells can synthesize cysteine from amino acid methionine through the transsulfuration pathway. A recent study revealed that N-Myc upregulated transsulfuration machinery leading to increased cellular cysteine through [16]. N-Myc transactivated several genes including cystathionine beta-synthase (CBS), S-adenosyl-L-homocysteine hydrolase (AHCY), Cystathionine gamma-lyase (CTH) to increase the cellular cysteine levels [16]. According to publicly available global ChIP seq data, Myc binds to the promoter of SLC7A11 [17]. Myc is a known target of E2F1 [18]. Overall, we have found that several ferroptosis markers are regulated by E2F1 including SLC7A11, ATF4, GPX4, ALOX5 and Myc. This complex role of E2F1 provides insight into how cancer cells can prevent oxidative stress while being highly proliferative.

## Results

### WT-RB and mutant phosho resistant RB (RBΔCDK) differentially regulate ferroptosis

**Figures 1:**
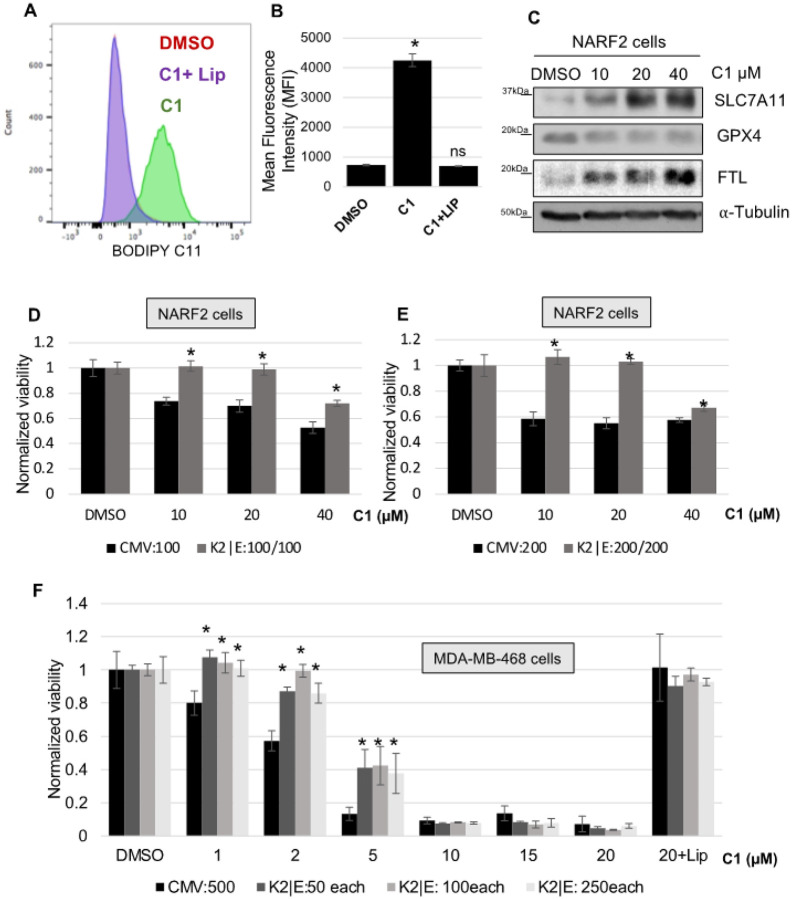
CDK2|cyclin-E co-expression inhibits ferroptosis. **(A)** NARF2 cells were treated with 20 μM CETZOLE-1 (C1) with or without Liproxstatin-1 (0.25 μM). One day later, lipid peroxides were measured using BODIPY C11 579/589. **(B)** Mean fluorescence intensity was plotted (n=3). **(C)** Western blot analysis shows the levels of ferroptosis marker proteins SLC7A11, GPX4, and ferritin light chain (FTL) in NARF2 cells upon 24h CETZOLE-1 treatment. **(D, E)** NARF2 or **(F)** MDA-MB-468 cells were transduced with CDK2/cycE adenoviruses at the indicated MOIs. Two days later, cells were treated with CETZOLE-1 at the indicated concentrations. Liproxstatin-1 (0.25 μM) co-treatment with highest dose of C1 in MDAMB468 cells confirms ferroptosis. Cell viability was determined 24h later using methylene blue. Represented western image is part of a blot. Original western blots are shown in **S. Figure 3.** (* p < 0.05)

We discovered a class of small molecules, “CETZOLE” that kill cells through ferroptosis. CETZOLE-1 induces lipid peroxidation in NARF2 (derived from osteosarcoma) cells and ultimately kills the cells. NARF2 cells are a CETZOLE-1 sensitive cell line derived from osteosarcoma U2OS cells. NARF2 cells contain IPTG inducible p14ARF that can be used to elevate p53. BODIPY C11 staining shows lipid peroxidation in NARF2 cells exposed to CETZOLE-1 (Fig. 1A, B). Liproxsatin-1 is an antagonist of ferroptosis which scavenges lipid peroxides and blocks cell death. Upon liproxstatin-1 co-treatment with CETZOLE-1, oxidized lipid level drop to the level of DMSO control. Further, western blot analysis showed corresponding change in proteins which are indicators of ferroptosis (Fig. 1C). For example, SLC7A11 and ferritin light chain (FTL) were increased upon treatment with CETZOLE-1. GPX4 downregulation was observed in cells exposed to CETZOLE-1 similar to Erastin, RSL3 and ML162 [19]. These observations confirm that CETZOLE-1 induces cell death through ferroptosis.

CDK2-cyclin E complex hyper-phosphorylates RB during late G1 phase and leads to S phase initiation. When RB is hyperphosphorylated, E2F1 is released and cells progress into S phase [20]. Elevated CDK2 activity protects from ferroptosis through an unknown mechanism (Fig. 1D, E) [7]. We speculated that CDK2 inhibits ferroptosis via phosphorylated RB. First, we overexpressed CDK2/cyclin E by means of adenovirus in MDA MB 468 cells (breast cancer cells with bi-allelic RB deletion) (Fig. 1F). Cell death was inhibited upon CDK2/cyclin E over expression in these cells. This could be due to other family members of RB; p107 and p130 which may inhibit ferroptosis, since their functions overlap each other family members. Otherwise, CDK2 could suppress ferroptosis through other substrate/s independent of RB family proteins.

In order to determine whether CDK2 activity suppresses ferroptosis through RB phosphorylation, we analyzed cells expressing a phospho-resistant RB mutant that cannot be phosphorylated by CDK2. RB has been reported to contain 14 CDK2 phosphorylation sites [9]. Previously a mutant of RB was generated by mutating all the 14 serine/ threonine residues into alanine (RBΔCDK) in Narasimha et al (2014). First, we tested the effect of expressing RBΔCDK on ferroptosis. Overexpression of WT-RB or CDK mutant RB (hereafter RBΔCDK / dCDK) might be expected to cause cell cycle arrest. Therefore, a transient system expression would be an appropriate approach for this study. WT-RB (RB) and RBΔCDK alleles [9] were inserted using AdEasy-1 system into recombinant adenoviruses [21]. AdTrack-CMV adenoviral backbone vector contains GFP as a mammalian expression reporter and we generated empty viruses containing GFP with no transgene as a control for the study.

**Figures 2:**
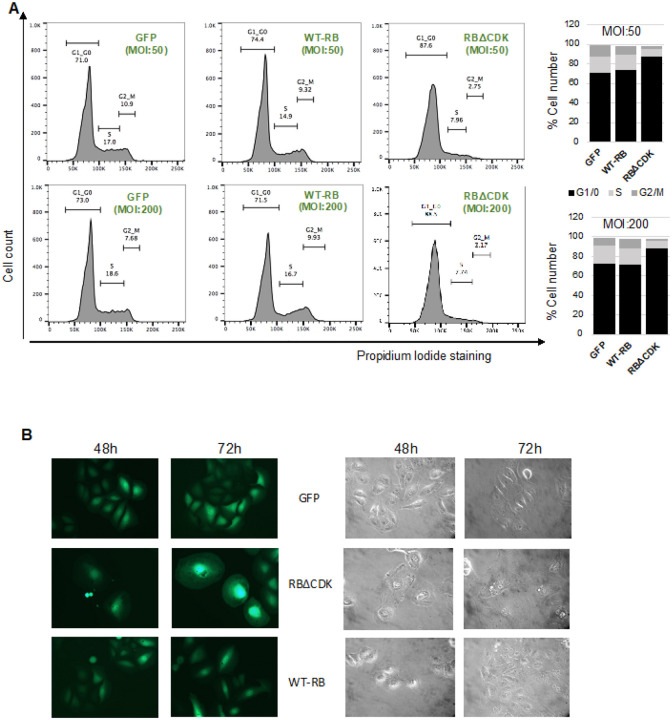
Characterization of adenoviruses expressing WT-RB and mutant RB (RBΔCDK). **(A)** Cell cycle phases of NARF2 cells were analyzed using propidium iodide staining 2 days after transduction with WT-RB, RBΔCDK and control GFP viruses. **(B)** Cell cycle phase percentage was represented in a bar chart. **(C)** Representative GFP and phase contrast images of NARF2 cells are shown upon infection with WT-RB, RBΔCDK and control GFP viruses.

Next, we characterized the RB expressing adenoviruses by western blotting and cell cycle profile analysis upon infecting the NARF2 cells. RBΔCDK expressing NARF2 cells were arrested in G0/G1 in 3 days (Fig. 2A). Because CDKs cannot phosphorylate RBΔCDK, E2F will not be released from this RB mutant leading to cell cycle arrest in G1 or G0. Consistent with this, RBΔCDK expressing cells had a senescence like morphology (large, flat and vacuole filled cells) confirming that the generated viruses are functional (Fig. 2B). Interestingly, WT-RB viruses did not cause G1 arrest in NARF2 cells (Fig. 2A). This may be due to higher levels of endogenous CDK activity in NARF2 cells to begin with. Thus, RB may still be phosphorylated leading to the release of E2F.

**Figures 3:**
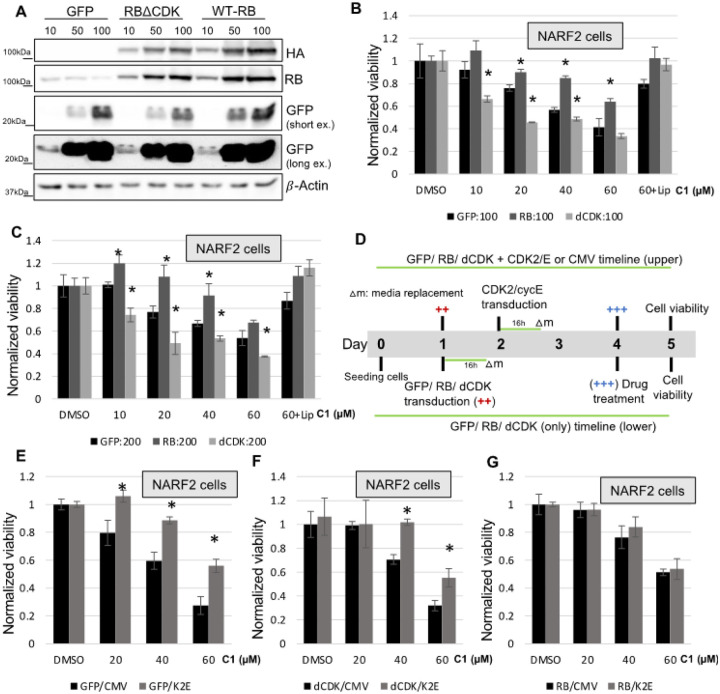
State of RB differentially regulates ferroptosis. **(A)** Western blot analysis depicts the expression of WT-RB, RBΔCDK and GFP proteins in NARF2 cells. After 3 days of transduction, cells were collected and analyzed by western blotting with antibodies against HA, RB, GFP and β-actin as a reference. **(B, C)** NARF2 cells were transduced with WT-RB, RBΔCDK and GFP viruses (MOI: 100 and 200). 72h later, the cells were treated with CETZOLE-1 (Liproxstatin-1 was cotreated with highest dose of C1). One day later, cell proliferation was measured by MTT assay. **(D)** Timeline diagram of sequential adenovirus transduction and ferroptosis measurement. **(E-G)** One day after the RB/GFP (MOI:100) virus transduction, NARF2 cells were again infected with CDK2|cycE adenoviruses (100 MOI each). 48h post transduction of CDK2|cycE, C1 was treated and cell viability was measured after 24h using MTT assay. Absorbance was measured at 540nm. Original western blots are shown in **S. Figure 4**. (* p < 0.05)

Western blot analysis confirmed the overexpression of WT-RB, RBΔCDK and GFP in NARF2 cells infected with the recombinant adenoviruses (Fig. 3A). Consistent with the previous results, WT-RB inhibited ferroptosis compared to the control GFP Remarkably, RBΔCDK enhanced killing as predicted in comparison to control GFP expressing cells (Fig. 3B, C). This could suggest that phosphoRB indeed suppresses ferroptosis. Next, we overexpressed CDK2/cycE in addition to WT-RB, RBΔCDK and control GFP Interestingly CDK2/cycE still inhibited ferroptosis in cells expressing RBΔCDK (Fig. 3D–G). These results suggest that CDK2/cycE may protect from ferroptosis independently of RB phosphorylation. However, CDK2/cycE did not inhibit ferroptosis in cells expressing WT-RB. This may be due to CDK2/cycE targeting RB for phosphorylation. All taken together, CDK2/cycE moonlights as a suppressor of ferroptosis independent of phosphorylating RB to regulate E2F activity.

#### Ferroptosis antagonism by E2F1

**Figures 4:**
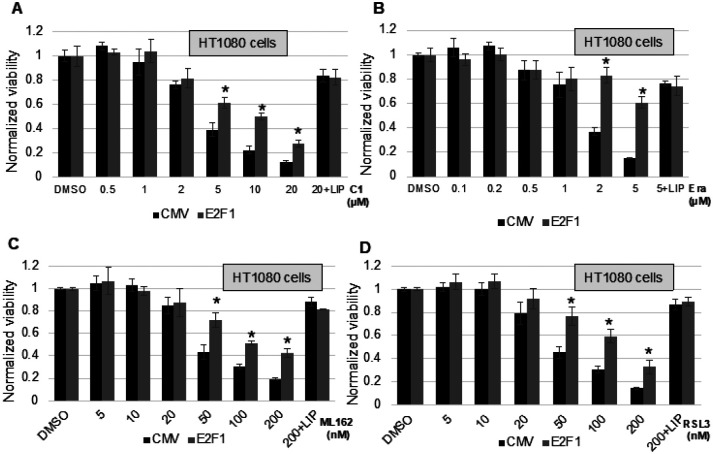
E2F1 protects from ferroptotic cell death. HT1080 cells were transduced with E2F1 adenoviruses for 2 days and then ferroptosis inducers CETZOLE-1 **(A)**, Erastin **(B)**, RSL3 **(C)** or ML162 **(D)** were added at the indicated concentrations. Highest dose of each inducer was co-treated with Liproxsatin-1 to confirm ferroptotic cell death. One day later, cell viability was measured using methylene blue staining. (* p < 0.05)

E2F1 is an essential transcription factor for cell cycle regulation. In most cancer cells, elevated E2F1 drives uncontrolled proliferation. We found that E2F1 protected from CETZOLE-1 induced ferroptotic cell death in part through upregulating p21. CETZOLE-1 induced cell death shows many of the hallmarks of ferroptosis such as accumulation of ferroptosis markers, iron dependency and complete rescue by antioxidants and iron chelators. Our results show that protection from E2F1 is not limited to CETZOLE-1 but also other ferroptosis inhibitors Erastin, RSL3 and ML162 (Fig. 4A–D). We overexpressed E2F1 in HT1080-LXSN cells (fibrosarcoma cells expressing empty vector LXSN, here onwards HT1080 cells) and 48h later the cells were treated with CETZOLE-1, Erastin, RSL3, or ML162. Upon E2F1 overexpression, the cells were significantly protected from these ferroptotic inducers. Moreover, cell death was rescued by lipid ROS scavenger, liproxstatin-1 when co-treated with the highest concentration of the above drugs. This confirm that cells are undergoing ferroptosis.

#### E2F1 transcriptionally upregulates SLC7A11

**Figures 5:**
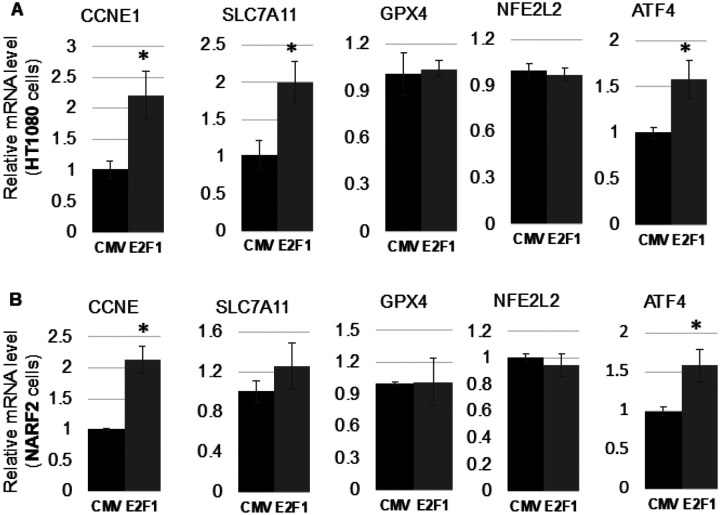
Expression of ferroptotic genes upon E2F1 overexpression. Adenovirus (100 MOI) expressing E2F1 or CMV control viruses were transduced for two days into **(A)** HT1080 or **(B)** NARF2 cells. Then, qPCR was performed to measure the mRNA levels of SLC7A11, NFE2L2, GPX4, ATF4, and CCNE1 as a reference of E2F1 target using SYBR green. (n=3 and * p < 0.05)

E2F modulates cell cycle progression, apoptosis, senescence and ferroptosis [22] [7]. To understand how E2F regulates ferroptosis we investigated candidate transcriptional targets. We performed an mRNA screen (primers are indicated in Table 1) of ferroptosis related genes by real time PCR (qPCR) and found that SLC7A11 is upregulated in HT1080 (human fibrosarcoma) cells upon E2F1 overexpression (Fig. 5A). A modest increase in SLC7A11 mRNA level in NARF2 cells was observed however, it was not statistically significant (Fig. 5B). Thus, the effect of E2F1 appears to be cell type dependent. Promoter analysis of SLC7A11 using UCSC genome data base (GSM935477) indicates that E2F1 binds to the promoter of SLC7A11 (**S.** Figure 2) [23]. Together, E2F1 can transcriptionally upregulate SLC7A11. SLC7A11 has been reported to be transcriptionally repressed by p53 [24]. This may possibly explain the results in NARF2 cells since E2F1 elevated higher levels of p53 in this cell line but minimal elevation in HT1080 cells. CCNE1(cyclin E) was used as a positive transcriptional control of E2F1 targets. Antioxidant master regulator (Nuclear erythroid factor 2 (Nrf2)) was reported to transactive several antioxidant genes including SLC7A11 [25] and GPX4 [26]. Therefore, we measured the mRNA level of NFE2L2 (encoding Nrf2) in both NARF2 and HT1080 cells after E2F1 overexpression (Fig. 5A, B). There was no change of NFE2L2 transcripts in response to E2F1 overexpression. We also measured mRNA levels of GPX4 in E2F1 overexpressed HT1080 and NARF2 cells (Fig. 5A, B). GPX4 transcripts were not altered upon E2F1 overexpression. This finding of GPX4 is consistent with unchanged NFE2L2 upon E2F1 overexpression since GPX4 is transactivated by NFE2L2.

SLC7A11 is modulated by the activation transcription factor (ATF/CREB) family. ATF has 6 family members. Amongst them, ATF4 upregulates SLC7A11 [27] [28]. On the other hand, ATF3 was reported to repress SLC7A11 [29]. Since SLC7A11 was upregulated, and E2F1 is mostly an activating transcription factor, we measured mRNA levels of ATF4 upon E2F1 overexpression. interestingly, ATF4 was upregulated in both HT1080 and NARF2 cells compared to CMV transduced cells (Fig. 5A, B). These results indicate that E2F1 may partly upregulate SLC7A11 through ATF4. Based on our qPCR and western blot results, E2F1 can transcriptionally upregulate SLC7A11.

**Figures 6:**
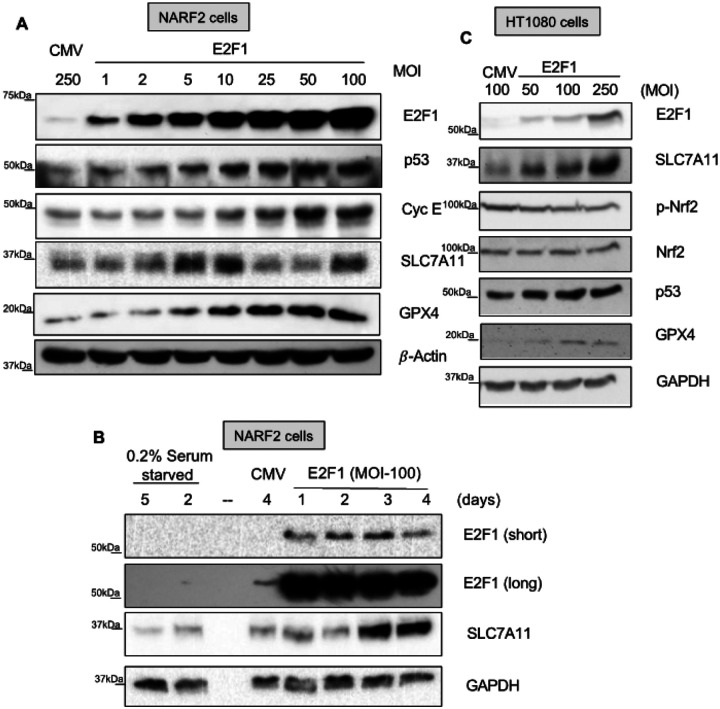
Western blot analysis after E2F1 overexpression. **(A, B)** NARF2 or **(C)** HT1080 cells were transduced with E2F1 or CMV adenoviruses at the indicated MOIs. **(A, C)** 2 days or **(B)** indicated times later cells were collected and immunoblotted for given proteins. Image C represents a part of western blot. Original western blots are shown in **S. Figures 5–7**.

Next, we performed western blot analysis to measure the protein level of SLC7A11. We performed E2F1 overexpression experiments in U2OS (human osteosarcoma) derived NARF2 cells and HT1080 cells by means of adenovirus (Fig. 6). in NARF2 cells, SLC7A11 depicts a biphasic behavior with an increase at the lower E2F1 levels followed by a decrease at higher E2F1 level (Fig. 6A). One of the possibilities for this oscillation may be the upregulation level of p53 in NARF2. E2F1 is known to stabilize p53 through upregulating p14ARF which prevents the degradation of p53 by MDM2. Notably, elevated p53 was observed in NARF2 cells upon E2F1 overexpression. This may possibly explain the trend of the SLC7A11 protein level as well as the mRNA level in NARF2 cells. Moreover, we noticed that SLC7A11 protein level was upregulated in a dose dependent manner in HT1080 where p53 level were less responsive to E2F1 overexpression. Interestingly, 3 and 5 days after viral transduction we observed a remarkable upregulation of SLC7A11 upon E2F1 overexpression in NARF2 cells (Fig. 6B). Further, serum starvation led to downregulation of SLC7A11. Temporal upregulation of SLC7A11 by E2F1 in NARF2 cells suggests that this regulation could be indirect. Next, we detected no change of Nrf2 proteins level after E2F1 overexpression in HT1080 cells similar to the mRNA level. Interestingly, E2F1 transduction leads to increased expression of GPX4 protein in both HT1080 and NARF2 cells compared to CMV control (Fig. 6A, C). This indicates that E2F1 may indirectly stabilize GPX4 protein since we observed no difference on mRNA. It is very interesting but not surprising that E2F1 regulates multiple ferroptosis related proteins. Further study is needed to confirm the mechanism of E2F regulation of these targets.

### E2F1 knockdown and downstream targets.

**Figures 7:**
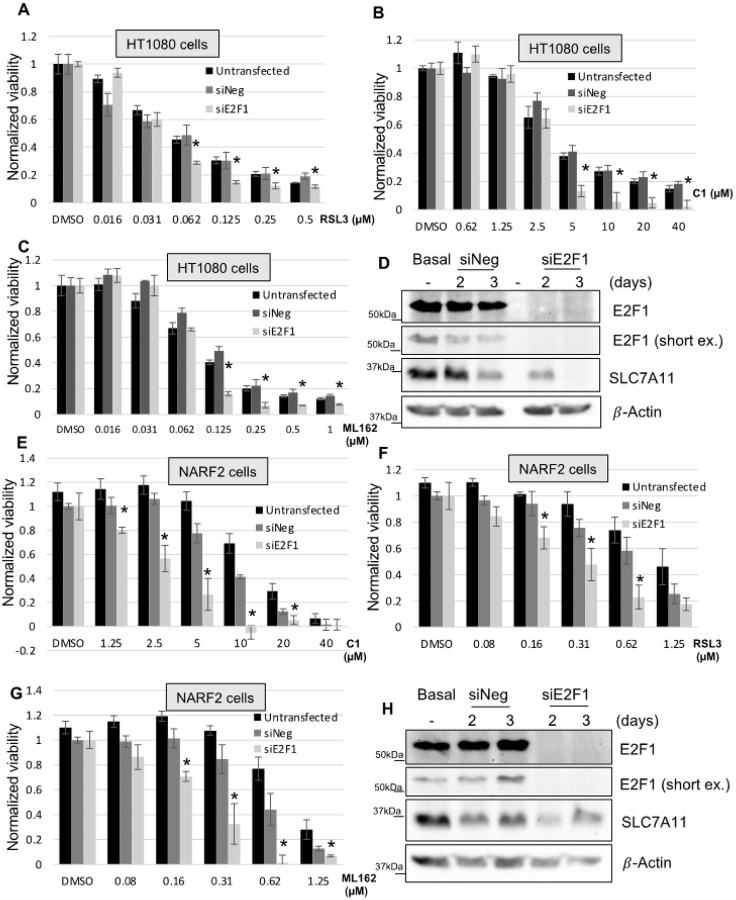
E2F1 knock down sensitizes sarcoma cells to ferroptosis. **(A-D)** HT1080 and **(E-H)** NARF2 cells were transfected with E2F1 siRNA or Negative control siRNA (siNeg/scramble) or no transfection (parental). Next day, cells were expanded into a 96 well plate and 3 days post transfection, cells were treated with CETZOLE-1, RSL3, or ML162 as indicated. Cell viability was measured using MTT assay one day later (**A-C; E-G**). Western blot analysis was performed to visualize the protein level (**D** and **H**). Original western blots are shown in **S. Figure 8**. (* p < 0.05)

**Figure 8:**
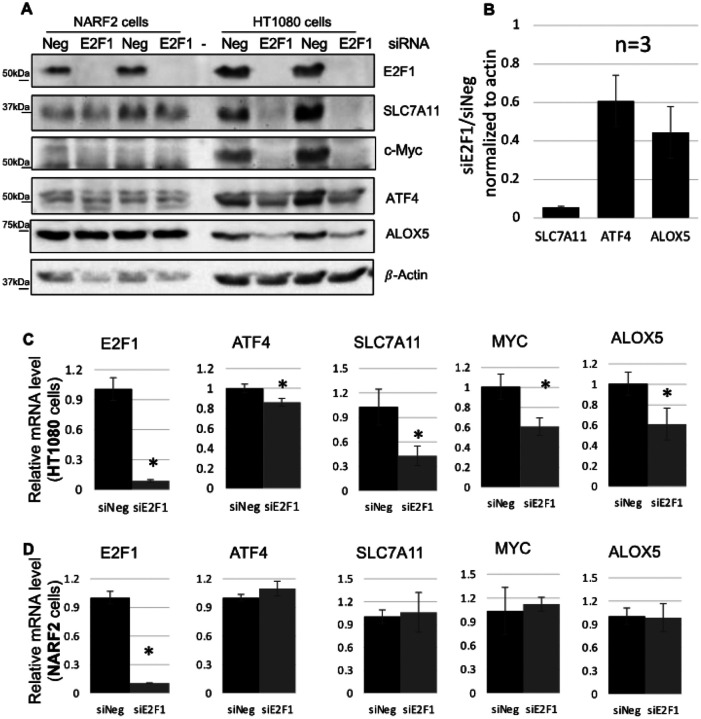
Cell type dependent regulation of SLC7A11, ALOX5, and ATF4 by E2F1. **(A)** Western blot analysis of the effect of E2F1 knock-down in HT1080 and NARF2 cells. Three days after transfection, protein levels of E2F1, SLC7A11, ALOX5, ATF4 were determined using β-actin as a loading control. **(B)** Bar graph shows the fold of protein levels of HT1080 cells in siE2F1 vs control siNeg. Transcripts level of the indicated genes were measured in both HT1080 **(C)** or NARF2 **(D)** cells after E2F1 or scrambled (siNeg) knock down for 3 days. Original western blots are shown in **S. Figure 9**. (* p < 0.05)

In order to confirm regulation of downstream targets by E2F1, we depleted E2F1 by siRNA in HT1080 cells and NARF2 cells. Western blot shows that more than 90% knock down of the protein was achieved (Fig. 7D, H)). As expected, both NARF2 (Fig. 7A–C) and HT1080 (Fig. 7E–G) cells become more susceptible to ferroptosis compared to control (siNeg/ scrambled) transfected cells or parental cells. Cell death in HT1080 cells was rescued by co-treatment with ROS lipid scavenger liproxstatin-1 and not with pan caspase inhibitor Z-vad (FMK) (**S.** Figure 1). Next, we measured the protein levels of SLC7A11, ATF4 and ALOX5 after E2F1 knock down (Fig. 8A, B). In HT1080 cells, SLC7A11 was repressed > 90% (n = 3) in 3 days. Downregulation of ATF4 (~ 40%) and ALOX5 (~ 55%) was also observed (Fig. 8B). Surprisingly, these protein levels were not altered in NARF2 cells. Next, we determined the mRNA levels in both HT1080 and NARF2 cells. SLC7A11, ATF4, Myc and ALOX5 were downregulated in HT1080 cells after E2F1 knockdown (Fig. 8C). Modest, but significant downregulation of ATF4 transcripts was observed in HT1080 cells. We have observed no change of the mRNA levels of these genes in NARF2 cells similar to no effects on the protein levels (Fig. 8D). We speculated that this differential regulation of the genes may be due to other family members of E2F1.

**Figure 9:**
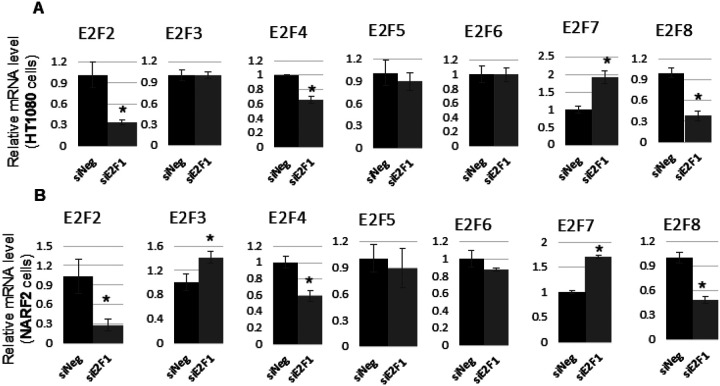
E2F1 knockdown affects its own family members. Transcripts levels of E2F family members were measured after E2F1 knockdown (3 days) in both **(A)** HT1080 and **(B)** NARF2 cells. (* p < 0.05)

E2F regulates its own family members [18]. We measured mRNA levels of other E2F family members by real time PCR analysis (Fig. 9). We found that mRNA levels of E2F2, E2F4 and E2F8 were downregulated, while no change was observed in E2F5 and E2F6 upon E2F1 knock down compared to negative control in both HT1080 (Fig. 9A) and NARF2 (Fig. 9B) cells. Interestingly, E2F3 transcript level was upregulated in NARF2 cells, but no change was detected in HT1080 cells. E2F2 and E2F3 are known to share the function of E2F1 in activating transcription [22]. Therefore, cell type dependent compensatory effect of E2F3 appears to distinguish NARF2 cells from HT1080 cells. This may possibly explain the differential expression of ferroptotic genes in NARF2 and HT1080 cells. These effects provide insight into E2F1 targets which vary depending on the cell type and participate in antioxidant defense mechanism.

#### C-Myc regulates SLC7A11

**Figure 10:**
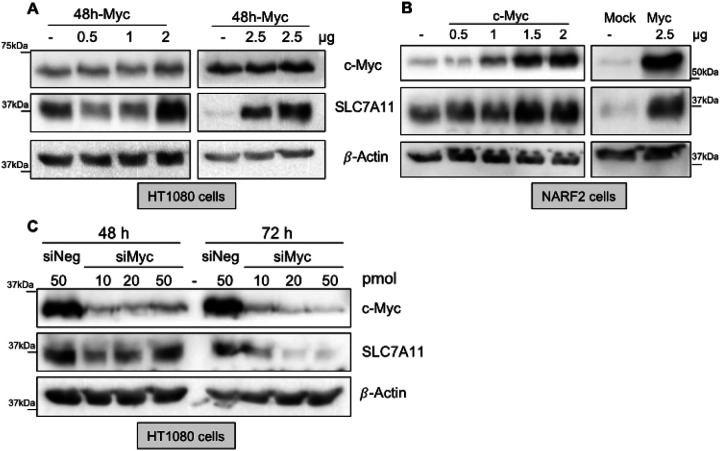
Transcription factor c-Myc upregulates SLC7A11. **(A)**HT1080 or **(B)** cells were transiently reconstituted with c-Myc and harvested 48h later. **(C)** HT1080 cells were transfected with siRNA against Myc (10–50 pmol) or non-targeted sequence (50 pmol, siNeg/ control) and the cells collected after 48h or 72h. **(A-C)** Harvested cells were analyzed by western blotting and probed for Myc, SLC7A11, and β-actin as a loading control. Different blots are shown together in right and left panel of A and B. Original western blots are shown in **S. Figure 10**.

Our assessment using the UCSC genome browser suggested that Myc binds to the SLC7A11 promoter (GSM935516) based on the genome wide ChIP seq data (**S.** Figure 2) [17]. Recently, N-Myc has been reported to promote cysteine addiction through transsulfuration [16]. We overexpressed c-Myc in HT1080 and NARF2 cells and found that SLC7A11 protein levels were upregulated (Fig. 10A, B). We have also noticed that endogenous level of c-Myc in HT1080 cells was notably higher than in NARF2 cells (Fig. 10A, B). Therefore, we overexpressed c-Myc in a dose dependent manner. Transfection of HT1080 cells with 2 and 2.5μg plasmid resulted in SLC7A11 transactivation (Fig. 10A). However, elevation of c-Myc level detected by western blotting was subtle. Overexpression of Myc can induce apoptosis [12]. Transfection of 3μg of c-Myc plasmids in HT1080 cells resulted in killing all the cells. This suggests that levels of Myc in HT1080 cells is almost in the upper limit of the threshold. Next, we overexpressed Myc in NARF2 cells and found a gradual increase of Myc as well as SLC7A11 (Fig. 10B). To confirm the results, we knocked down Myc using different concentrations of siRNA in HT1080 cells (Fig. 10C). As expected, Myc knock down led to repression of SLC7A11 protein in 3 days. In 2 days, a modest suppression was observed in the levels of SLC7A11 protein. Based on our results and previous reports, Myc transcriptionally activates SLC7A11. Furthermore, in E2F1 knockdown experiment, Myc mRNA level was not altered in NARF2 cells and downregulated in HT1080 cells. This further supports that differential compensatory effect of E2F3 when E2F1 is depleted in NARF2 cells rather than HT1080 cells.

#### SLC7A11 overexpression protects from multiple ferroptotic signals

**Figure 11:**
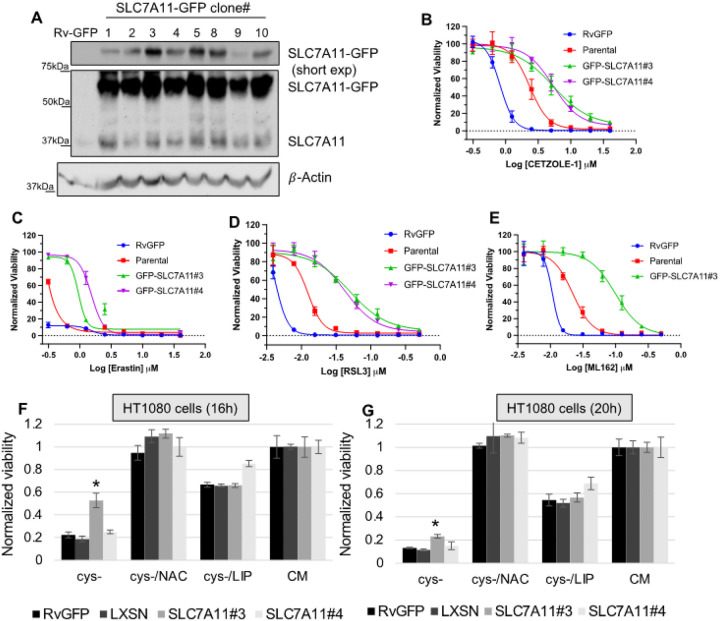
Overexpression of SLC7A11 protects from various ferroptotic inducers. SLC7A11-GFP fusion protein was stably expressed in HT1080 cells by retroviral transduction and individual cell lines were generated. Control GFP expressing cell line was generated using lentivirus (Rv-GFP#1). **(A)** Western blot analysis demonstrates the level of endogenous and ectopic SLC7A11-GFP SLC7A11-GFP clones #3 and #4 were tested for ferroptosis sensitivity using ferroptosis inducers: **(B)** CETZOLE-1, **(C)**Erastin, **(D)** RSL3, **(E)** ML162 and **(F G)** cystine deprivation. Parental and Rv-GFP cells were used as control cells. Cells were deprived of cystine for 16 or 24h. All other inducers were treated for 3 days and cell viability was measured by methylene blue staining. Original western blots are shown in **S. Figure 11**. (* p < 0.05)

To study the effect of SLC7A11 in ferroptosis, we generated stable cell lines of HT1080 cells overexpressing SLC7A11-GFP fusion protein by means of retroviral transduction. SLC7A11-GFP plasmid was a generous gift form Alec Kimmelman (New York University School of Medicine) [30]. As a control, a GFP expressing HT1080 cell line was generated by retroviral transduction (HT1080 Rv-GFP). Western blot analysis shows the independent cell lines expressing SL7A11-GFP and control Rv-GFP (Fig. 11A). Clone #3 and #4 were chosen to measure ferroptosis sensitivity of different ferroptosis inducers. We also tested if SLC7A11 over expression protects from CETZOLE-1 (Fig. 11B), Erastin (Fig. 11C), RSL3 (Fig. 11D) and ML162 (Fig. 11E). As expected, SLC7A11 clones #3 and #4 inhibited cell death from both SLC7A11 inhibitors and GPX4 inhibitors compared to control HT1080 Rv-GFP cells as well as parental cells (HT1080). We have also noticed that HT1080 Rv-GFP cells were more sensitive to all the tested ferroptosis inducers compared to parental cells.

Next, we tested ferroptosis sensitivity of the SLC7A11-GFP expressing HT1080 cell lines upon cystine deprivation. Interestingly, clone #3 was protected from cell death ~ 50% at 16h (Fig. 11F) and ~ 20% at 24h (Fig. 11G). However, clone #4 was not protected from cystine deprivation. Notably, the expression level of SLC7A11 in clone #3 is higher than clone #4. Increased level of SLC7A11 is expected to result in elevated SLC7A11 activity. Hence, intracellular cystine pool would be higher upon overexpression of SLC7A11 in clone #3 resulting in delayed death upon cystine deprivation. Cell death was rescued by Liproxstatin-1 and N-acetyl cysteine (NAC). NAC is a stable form of cysteine and transported inside the cells independent of system Xc^−^. Taken together, SLC7A11 overexpression protected the cells from both SLC7A11 and GPX4 inhibitors. SLC7A11-GFP likely leads to the elevated intracellular glutathione (GSH) through a higher level of Xc^−^ transporter activity. Glutathione is an antioxidant itself apart from being a co-factor of GPX4 enzyme. GSH may be quenching ROS accumulation even in cells exposed to GPX4 inhibitors (CETZOLE-1, RSL3 and ML162) resulting in enhanced survival.

#### SLC7A11 upregulation occurs in response to both GPX4 and system Xc^−^ inhibition

**Figure 12:**
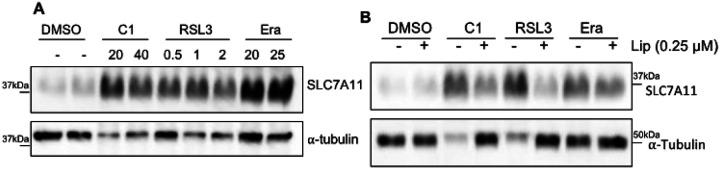
SLC7A11 upregulation upon ferroptosis induction is not limited to system Xc- inhibitors. NARF2 cells were treated with CETZOLE-1 (C1), Erastin, or RSL3 at the indicated concentration for 24h. Western blot was performed, and membranes were probed with antibodies against SLC7A11, GPX4, FTL, and -tubulin as a loading control. Original western blots are shown in **S. Figure 12.**

System Xc^−^ is comprised of two subunits, SLC7A11 and SLC3A2 (4F2hc). In 2004, Sato et al had reported that system Xc^−^ activity (by cystine uptake), mRNA levels (by luciferase reporter assay of SLC7A11) were upregulated upon deprivation of several amino acids including Cysteine and Arginine in NIH3T3 cells (mouse fibroblasts) [31]. Western blot analysis showed that both SLC7A11 and SLC3A2 subunits were upregulated [31]. Further, they showed that ATF4 was involved in SLC7A11 expression in the same study [31]. In 2008, Lo et al reinforced that both SLC7A11 and SLC3A2 subunits have been transcriptionally upregulated upon inhibition of the system Xc^−^ transporter (cystine deprivation) by qPCR and western blot analysis in several pancreatic cancer cell lines [32]. Several studies have implied that the upregulation of system Xc^−^/ SLC7A11 is a characteristic features of system Xc^−^ inhibitors [33] [34]. Therefore, we performed western blot analysis in NARF2 cells after treatment of CETZOLE-1, Erastin and RSL3 without (Fig. 12A) and with liproxstatin-1 (Fig. 12B). Interestingly, SLC7A11 upregulation was observed with both system Xc^−^ inhibitors (Erastin and CETZOLE-1) and GPX4 inhibitors (RSL3 and CETZOLE-1). The initial work on system Xc^−^ inhibition and SLC7A11 upregulation was reported before ferroptosis was discovered in 2012 [3]. In Dixon et al (2012), SLC7A11 mRNA levels were not changed when beta (β)-mercaptanol (βME) was co-treated with erastin but mRNA levels were still upregulated with DFO or Ferrostatin-1 cotreatment with erastin in HT1080 cells for 6h [3]. βME cleaves the disulfide bond of the cystine resulting in two cysteine molecules which bypass Xc^−^ to enter the cell. βME also act as an antioxidant. Hence, cells cotreated with βME and erastin are not deprived of cysteine. However, Ferrostatin or DFO cotreated with erastin cells are still deprived of cysteine even though they are rescued from cell death. Therefore, SLC7A11 mRNA level was not changed when the cells were cotreated with Erastin and βME [3]. Our western blot shows, SLC7A11 level was lower than cells co-treated with liproxsatatin-1 and CETZOLE-1, RSL3, or Erastin than the cells exposed with only CETZOLE-1, RSL3, or Erastin (n = 3). However, SLC7A11 levels were higher in NARF2 cells when cotreated with liproxstatin-1 and ferroptosis inducers compared to the DMSO treated cells. This suggests that elevated ROS may have significantly contributed to the SLC7A11 upregulation. ROS accumulation can lead to Nrf2 signaling and Nrf2 can upregulate SLC7A11 which may be a part of the reason [34]. Overall, our results and previous finding [19] suggests that SLC7A11 upregulation is response to ROS accumulation during ferroptosis but not limited to system Xc^−^ inhibition.

## Discussion

Ferroptotic cell death was identified in 2012 and the regulatory mechanism still needs to be unraveled. Our interest in cell cycle regulators and ferroptosis emerged from the fact that cancer cells are more proliferative and metabolically active compared to normal cells. Therefore, antioxidant defense system in cancer cells would be highly upregulated. We recently uncovered that p53/p21/CDK/RB/E2F pathway proteins play differential and opposing roles in ferroptosis. For example, p53 enhances ferroptosis while p21 suppresses ferroptosis. Likewise, both RB and E2F1 inhibit ferroptosis while RB is known to bind and inactivate the function of E2F1. The current study further explores how RB and E2F regulate ROS accumulated cell death.

We have previously shown that elevated CDK2 activity inhibits ferroptosis. CDK2 primarily phosphorylates RB and results in release of E2F1. Recent studies revealed that RB is monophosphorylated during G1 and some of the monophosphorylated RB species (S811 and T826) regulated the expression of oxidative phosphorylation (OXPHOS) genes. We hypothesized that CDK2 could protect from ferroptosis through phosphorylating RB and certain phosphoRB species may be responsible for inhibition of ferroptosis. In order to address the role of RB phosphorylation in ferroptosis, we generated adenoviruses expressing WT-RB and RBΔCDK where all the 14 phosphorylation sites were mutated to phospho-resistant alanine residues. Consistent with the previous findings, WT RB suppressed ferroptosis. Remarkably, the cells expressing RBΔCDK become more susceptible to ferroptosis. This indicates that RB phosphorylation may potentially play a role in resistance to ferroptosis. During senescence, intracellular ROS levels are higher than normal cells [35] [36]. This implies that senescent cells may withstand relatively higher ROS levels. Therefore, RBΔCDK induced senescence may have enhanced ferroptosis, although we have no direct evidence for this explanation. Further studies are needed to find the exact mechanism.

Inhibition of ferroptosis by CDK2 in RB null MDA-MB-468 cells indicates that CDK2 works independent of WT-RB. However, this does not exclude effects of CDK2 on RB family members. Overexpression of RBΔCDK and CDK2/cycE in NARF2 cells suggests that CDK has other targets independent of RB family proteins. Interestingly, CDK2/cycE co-expression with WT-RB did not change the ferroptosis sensitivity in NARF2 cells. This result suggests that CDK2 likely phosphorylates RB family proteins as primary targets. All taken together, ferroptosis inhibition by CDK2 in RBΔCDK expressing NARF2 cells indicates that CDK2 moonlights to protect from ferroptosis cell death independent of cell cycle.

In our previous study, we demonstrated that E2F1 protects from CETZOLE-1 induced ferroptosis in NARF2 and HT1080 cells. Our present study indicates that the suppressing effect of E2F1 in ferroptosis is not limited to CETZOLE-1. Rather, E2F1 protects from multiple ferroptosis inducers including Erastin, RSL3 and ML162. We have previously determined that E2F1 partially protects from ferroptotic cell death through CDK inhibitor, p21. In order to identify other targets of E2F1 responsible for ferroptosis inhibition, we performed a gene expression screen of several ferroptosis candidates by qPCR analysis. Interestingly, we found that the mRNA levels of SLC7A11 was upregulated in HT1080 cells after E2F1 overexpression. SLC7A11 proteins were detected very similar to the trend of mRNA levels. Interestingly, we noticed a biphasic behavior of SLC7A11 upregulation in NARF2 cells. This may be due to upregulated p53 or other cell type dependent targets of E2F1. Transcription factors ATF4 and Nrf2 can upregulate SLC7A11 and block ferroptosis. Thus, we measured the mRNA levels of these genes and found NFE2L2 levels were unchanged but ATF4 was upregulated by E2F1. Interestingly, the levels of GPX4 protein was increased upon E2F1 overexpression. However, GPX4 mRNA levels were not altered, suggesting that E2F1 regulates GPX4 post-transcriptionally. Moreover, we identified a pro ferroptotic gene ALOX5 as another target of E2F1.

Our E2F1 knockdown experiments in HT1080 and NARF2 cells indicates that both cell types become more prone to ferroptosis upon depletion of E2F1. Furthermore, SLC7A11, ATF4, ALOX5 and c-Myc mRNA were reduced after E2F1 siRNA knockdown in HT1080 cells. Surprisingly, these proteins or mRNAs were not altered in NARF2 cells after 3 days of E2F1 knockdown. Nevertheless, we noticed a slight downregulation of SLC7A11 at 48h post siRNA transfection in NARF2 cells. After 3 days of E2F1 knockdown in NARF2 cells, SLC7A11 levels were elevated almost to the basal level even though E2F1 was still absent (> 90%). This oscillatory observations in NARF2 cells could be due to cell type dependent compensation of other E2F family members. Our screen of other E2F family members upon E2F1 knockdown in HT1080 and NARF2 cells showed how E2F1 differentially affect other members of the same family. Notably, we found that E2F3 was upregulated in NARF2 cells but remained unchanged in HT1080 cells. This potentially explains the differences between these two cell lines. However, it remains elusive how NARF2 cells becoming more sensitive to ferroptosis upon knockdown of E2F1. This suggests that E2F1 has various targets to overcome oxidative stress depending on the cell type.

WT-RB and RBΔCDK experiments shows that the phosphorylation state of RB can impact the sensitivity to ferroptotic cell death. Future studies will reveal the exact mechanism of action. Since SLC7A11 does not contain a consensus sequence of E2F binding site (our unpublished data), we speculated that E2F1 may indirectly transactivate SLC7A11. Based on our qPCR results, we found ATF4 and c-Myc were upregulated with E2F1 overexpression. SLC7A11 upregulation by E2F1 can be part of the E2F and ATF4 or Myc axis. Altogether, we identified several pro and anti-ferroptosis genes that are modulated by E2F1 including SLC7A11, GPX4, ATF4 (anti) and MYC, ALOX5 (pro).

## Methods

### Cell Lines and Culture Conditions.

Cell lines were cultured in a humidified atmosphere containing 10% CO_2_ in Dulbecco’s Modified Eagle’s Medium (Mediatech, Inc.) supplemented with 10% fetal bovine serum (Atlanta Biologicals). Cell types used; HT1080-LXSN (human fibrosarcoma cells expressing empty vector LXSN), NARF2 (osteosarcoma), MDA MB 468 (breast cancer).

For adenoviral transduction, 2000–4000 NARF2 or 2000–3000 HT1080 cells or 8,000 MDA MB 468 cells were plated per well into 24 well plates throughout the study. Next day, cells were infected with viruses and one day later, fresh media was replaced. Drugs were added 48–72h post adenoviral infection. Cells were stained 1–3 days later with a saturated solution of methylene blue in 50% ethanol. Plates were rinsed and retained dye quantified by spectrophotometry. Absorbance was normalized to DMSO and given as 1 or 100% for cell viability. Some of the experiments were carried out with MTT assay (indicated at the appropriate places). We have recently shown that, in the context of ferroptosis, methylene blue staining results are very similar to MTT assay [6]. Results are representative of at least two independent experiments. Statistical significance was assessed using the students t-test. All commercially available chemicals were obtained from Cayman Chemicals unless otherwise noted. Novel compound CETZOLE 1 was synthesized as we have described in [4].

### MTT assay.

12 mM MTT (Goldbio) stock solution (10 X) was prepared in 1X PBS. For 24 well plates, at the harvesting time, media was replaced with 300 μl fresh media. 30 μl of MTT stock solution was added and incubated at 37°C. After 3 hours, equal amount of MTT lysis solution (0.01 N HCl in 10% SDS) was added and incubated for 30 minutes. Finally, absorbance was measured at 540 nm.

### Generation of RB adenoviruses and purification.

First generation of RB adenoviruses were constructed and generated using AdEasy1 system. WT RB and RBΔCDK ORF were cloned from the plasmids constructed in Dowdy lab using the following primers (5″ to 3″); forward primer-ACCGTCGACGCGGCCGCCACCATGTACCCATACG and reverse primer-TAGGCTCGAGCGGCCTCATTTCTCTTCCTTGTTTG. Next, the PCR amplicon was inserted into Pmel and EcoRI linearized shuttle vector AdTrack-CMV was a generous gift from Bert Vogelstein (Addgene plasmid # 16405; http://n2t.net/addgene:16405 ; RRID:Addgene_16405) using infusion cloning kit (Takara) [37]. Then, colonies were isolated, plasmid extracted by alkaline lysis method and screened by restriction digestion. Sequences were confirmed with the following primers: universal LNCX forward primer AGCTCGTTTAGTGAACCGTCAGATC, forward primers at different sites of RB plasmids GCTATGTGTCCTTGACTATT (starts at 685bp), CAACTGCACAGTGAATCC (at 1215bp), GTGATCGAAAGTTTTATCAAAGC (at 1628bp) and CCTCGAAGCCCTTACAAG (at 2416bp).

Homemade electrocompetent cells (AdEasier) were cultured by transformation of AdEasy-1 plasmid into BJ5133 strains. pAdEasy-1 (Addgene plasmid # 16400 ; http://n2t.net/addgene:16400; RRID:Addgene_16400) and BJ5133 strains (Addgene plasmid # 16398) were generous gift from Bert Vogelstein [37]. GOI containing (WT-RB or RBΔCDK) or empty (GFP control) shuttle vectors were linearized using PacI enzyme and electroporated into AdEasier cells (2500V, 2.5Ω and 25μF). Transformed cells were cultured, individual colonies were isolated and screened by PacI restriction digestion. Restriction mapping confirmed individual plasmids were transfected into HEK293 cells for 10–14 days. Viruses were released from the cells by 3 freeze thaw cycles and collected. Then, individual colonies of viruses were insolated using plaque purification method as described below.

HEK293 cells were seeded into a 6 well plate at 50–70% confluence per well. Next day, cells were infected with 1 ml of culture media with appropriate dilution from the virus stock (1:1000 to 1:10,000). Six hours later, media was gently replaced with 3ml complete media containing 5% agarose (preheated at 44°C). The plate was allowed to solidify at RT in a biosafety cabinet and incubated at 37°C for 7–10 days. Individual plaques were isolated and used to infect 24 well plates and confirmed by western blot analysis.

Adenoviruses (confirmed clones) were propagated and purified by discontinuous CsCl gradient method. CsCl gradient was created by adding 6ml of 4.0M CsCl (67g CsCl in 100ml of 10mM HEPES) at the bottom layer, 9ml of 2.2M CsCl (38g CsCl in 100ml of 10mM HEPES) and 15ml of adenoviral supernatant at the top. Tubes (Beckman #344058) were ultracentrifuged at 34, 500 rpm in Type70 Ti rotor (Beckman ultracentrifuge). Virus band was obtained between 2.2M and 4M CsCl and collected by puncturing with a 18G needle. Collected viruses were dialyzed against 10% glycerol containing 1X PBS at 4°C overnight twice. Lastly, the virus stocks were dialyzed by 5% glycerol containing 1X PBS at 4°C for 4h. Virus titers were determined by infecting HEK293 cells by serially diluted virus stocks. GFP reporter expression was used to calculate the virus titer.

### SLC7A11-GFP overexpression in HT1080 cells.

Phoenix retroviral packaging cells were plated to be 90% confluency on the day of transduction into a 6 well plate. The cells were transfected with SLC7A11-GFP plasmid, a gift from Alec Kimmelman [30] or control Rv-GFP plasmid (2.5 μg) using lipofectamine 3000 (Life technologies) according to manufacturer’s instructions. After 16hrs, media was replaced. Generated retroviruses were collected 48hrs post transfection. HT1080 cells from 24 well plates were infected with 100 μl of 48hr-viruses separately along with polybrene (4μg/ml). Next day, cells were expanded into 10 cm plates separately. SLC7A11 -GFP cells were grown in culture media containing neomycin (400μg/ml). Since control GFP cells had no antibiotic resistant genes, individual colonies were selected by GFP expression using a fluorescence microscope. Moreover, collected Rv-GFP clone was sub-cultured to isolate individual clones again. After 10–14 days, GFP or GFP- SLC7A11expressing colonies were selected, expanded, grown and confirmed by western blot analysis.

### Western Blotting.

Cells were harvested by scraping and lysed in a buffer solution containing: 50 mM Tris (pH 7.4), 150 mM NaCl, 0.5% NP-40, 1 mg/ml aprotinin, 2 mg/ml leupeptin, 1 mg/ml pepstatin A, 1 mM DTT, 0.1 M phenylmethylsulfonyl fluoride (PMSF), 1 mM sodium fluoride and 1 mM sodium vanadate for 20 min on ice. insoluble debris was removed by centrifugation at 16,000 g for 20 min at 4°C. Equal amounts of protein for each sample (determined using BCA protein assay kit - Pierce) were separated by sodium dodecyl sulfate polyacrylamide gel electrophoresis (SDS-PAGE). Gels were transferred to polyvinylidene difluoride membranes (Millipore), blocked in a solution containing 5% (w/v) non-fat dry milk dissolved in PBST [PBS containing 0.05% (v/v) Tween 20], and probed with antibodies as indicated. For phospho antibodies, membranes were blocked, or primary antibodies were diluted in 5% (w/v) bovine serum albumin in tween containing tris buffered saline.

Antibodies were generally diluted in blocking solution at 1:1000. Primary antibodies recognizing SLC7A11 (cell signaling technologies, CST#12691S), ATF4 (Abclonal #A0201), c-Myc (CST #9402S), FTL (Proteintech #10727–1-AP), RB (CST#9309S), HA (CST #3724S), cyclin-E (BD pharmingen #BD-51–1459GR), p21 (Santa Cruz #C-19/HRP), p53 (Santa Cruz #D0–1/HRP), E2F1 (Santa Cruz #56661), Nrf2 (Abclonal #A0674), p-Nrf2 (S40)(Abclonal #AP1133), GPX4 (Abclonal #A11243), GFP (Santa Cruz #9996/HRP), ALOX5 (Abclonal#A2877)), CDK2 (Santa Cruz #M2), -Tubulin (Sigma #T5168), GAPDH (Abclonal #AC002) and *β*-Actin (Abcam #3280) were generally incubated at 4° C overnight. Signals were detected using horse-radish peroxidase conjugated secondary antibodies (Biorad) and enhanced chemiluminescence (Biorad). Western blot images were mostly taken by a chemi doc and the digital images were analyzed using ImageJ software.

### Lipid ROS measurement.

NARF2 pBpuro cells were seeded as 5 × 10^5^ cells into a 9cm plate. Next day, the cells were treated with 20 μM CETZOLE-1 along with BODIPY 665/676-C11 dye (0.5 μM) (Thermofisher). 24 h post treatment, cells were collected by trypsinization, washed with 1x PBS and resuspended in 2% FBS containing 1x PBS. Cells were analyzed in PE channel using a BD LSR Fortessa FACScanner. 20, 000 events per condition was analyzed from 3 independent samples. The experiments were performed in duplicate (n = 6). Collected data were processed with FlowJo v10 software.

### siRNA transfection.

NARF2 and HT1080 cells were seeded in 6 well plates at a density of 2 × 10^5^ and 3 × 10^5^ respectively. Next day, the cells were transfected by 50 pmol of siRNAs against E2F1 or 50 pmol non-specific (scrambled/ siNeg) or Myc (10–50pmol) using Lipofectamine RNAi Max reagent according to the manufacturer’s instructions (Invitrogen life technologies). One day later, cells from multiple wells were expanded into 96 well plate for ferroptosis sensitivity assay or 6cm/ 9cm plates for western blot/ RNA extraction. siRNAs were obtained from Life technologies siE2F1 (cat#4427038), siMyc (cat#4427037) and siNeg (cat#4390843)

### Cell cycle analysis by PI staining.

NARF2 cells were seeded at 1 × 10^5^ cells per 6cm plate. After one day, cells were transduced with GFP WT-RB and RBΔCDK viruses at 50 and 200 MOIs. The Next day, media was replenished with fresh media. Three days post transduction, the cells were harvested by trypsinization, washed in 1X PBS and resuspended in 400μl of 1X PBS containing 2% FBS (PBSP). Then, cells were fixed with 700μl of pre-chilled 100% ethanol by adding drop wise (while vortexing) and kept at −20°C for 30 minutes. Cells were spun down and resuspended in 1ml of 2% PBSP The cells were treated with RNase-A at 50μg/ml for 30min at 37°C. Cells were collected, stained with Propidium Iodide dye (10μg/ml) and analyzed by flow cytometry.

#### Transient c-Myc overexpression

NARF2 (2 × 10^5^) or HT1080 (4 × 10^5^) cells were plated into 6 well plates. The next day, cells were transfected with indicated amount of c-Myc plasmid (0.5–2.5μg) using Lipofectamine LTX Plus reagent (Life technologies). One day later, cells were expanded into 6cm or 7cm plate according to the experiment. 48h later post transfection, cells were harvested and analyzed by western blotting. As a transfection control, cells were transfected with a GFP reporter plasmid. We observed > 90% transfection efficiency by GFP positive cells.

#### Real time PCR (qRT-PCR)

Cellular RNA was extracted using TRIzol (Invitrogen Life technologies) method according to the manufacturer’s protocol. 1μg RNA was used to synthesize cDNA by reverse transcription reaction using oligo-dT primer according to NEB protocol (MuLV RT kit #BO2535). qPCR reactions were performed using iTaq Universal SYBR Green Supermix (Bio-Rad #1725121) and run it on Biorad CFX96 machine. Relative mRNA expression level was normalized to GAPDH and quantified using comparative C_T_ method (ΔΔC_T_). n = 6, biological triplicates and technical duplicates. Primer sequences for the genes are shown in Table 1.

### Determining MOI of adenovirus (non-GFP).

HEK293 cells were plated in 10 cm plate and next day infected with second-generation recombinant adenoviruses. Generated viruses were collected 2–3 days later from both the supernatant and cells by freeze-thawing process three times (frozen in dry ice and thawed at 37°C). To determine viral titers, HEK293 cells were seeded in 96 well plates and next day, the virus suspension was diluted at 1:100. Later, 10μl virus supernatant was added to cells in 90μl culture media. One week later, cytopathic effect (CPE) was assessed by phase contrast microscopy. Alternatively, infected cells were fixed in 100% methanol at −20°C for 20 min. Cells were blocked by 1% PBSP One hour later, cells were probed with FITC conjugated Hexon antibody at 1:5000 in blocking buffer for 1 h. Cells were washed 3X with PBSP and infected cells visualized by fluorescence microscopy using an EVOS microscope. Titers determined using CPE or hexon immunofluorescence were similar.

#### Statistical analysis

Statistical significance was determined (* p < .05) by Student’s t test. Measurements of cell viability involved the preparation of triplicate samples, used to calculate averages and standard deviations. All cell viability studies were repeated in this manner at least once (total n ≥ 6). IC50 curves of SLC7A11-GFP clones were plotted using GraphPad Prism 9.0. Gene expression (mRNA) level was determined using comparative C_T_ method (ΔΔC_T_). n = 6, biological triplicates and technical duplicates.

## Figures and Tables

**Table 1:** Primer sequences for real time-PCR

Gene (Human)	Oligo nucleotide sequence
ATF4	Fw: AAACCTTACGATCCTCCTGGAG
	Rev: TGGCTGCTGTCTTGTTTTGC
CCNE1	Fw: ATGGCCAAAATCGACAGGAC
	Rev: AGGCTTGCACGTTGAGTTTG
SLC7A11	Fw: GGCAGTGACCTTTTCTGAGC
	Rev: TCATTGTCAAAGGGTGCAAA
GPX4	Fw: TGGTTAACCTGGACAAGTACCG
	Rev: AAACCACACTCAGCGTATCG
C-MYC	Fw: CCTGGTGCTCCATGAGGAGAC
	Rev: CAGACTCTGACCTTTTGCCAGG
GAPDH	Fw: GTCGGAGTCAACGGATTTGG
	Rev: TGGAATTTGCCATGGGTGGA
ALOX5	Fw: TCGATGCCAAATGCCACAAG
	Rev: TGAAGCGGTTGATGAACAGG
E2F1	Fw: CCATCCAGGAAAAGGTGTGAAA
	Rev: GGTGATGTCATAGATGCGCC
E2F2	Fw: ATGACATCACCAACGTGCTG
	Rev: CTGGTGGGGTCTTCAAACATTC
E2F3	Fw: AGGGCTCTCTTACACCGCACT
	Rev: AAATGCCACTCACACAATGGG
E2F4	Fw: GTCACAGAGGACGTGCAGAA
	Rev: GCCAAGAGGGTATCTCCAGC
E2F5	Fw: CCATTCAGGCACCTTCTGGTAC
	Rev: AGCAGCACATGGATAGGTCCTG
E2F6	Fw: GCGGAGAGTGTATGACATCACC
	Rev: GTCAGAAAGTTCCTCCTGTAGCT
E2F7	Fw: GACAGACAGCAAGCGGAACC
	Rev: GGGTCGGAATAGTCCCTTTTTCT
E2F8	Fw: GAGGCTCAAAGAGGGCAAGCAT
	Rev: ATGAGCACTGCGTGAGAGGGAT

## Data Availability

All data generated or analyzed during this study are included in this published article [and its supplementary information files]. Raw data sheets are available upon request.
